# IR-61 Improves Voiding Function *via* Mitochondrial Protection in Diabetic Rats

**DOI:** 10.3389/fphar.2021.608637

**Published:** 2021-04-14

**Authors:** Jianwu Wang, Linyong Dai, Xiaofeng Yue, Chongxing Shen, Tong Li, Lei Long, Yi Zhi, Yawei Wang, Gufang Shen, Chunmeng Shi, Yunsheng Liu, Qiang Fang, Weibing Li

**Affiliations:** ^1^ Department of Urology, The Third Affiliated Hospital (Gener Hospital) of Chongqing Medical University, Chongqing, China; ^2^ State Key Laboratory of Trauma, Burns and Combined Injury, Institute of Rocket Force Medicine, Third Military Medical University, Chongqing, China

**Keywords:** bladder smooth muscle cells, diabetic bladder dysfunction, IR-61, mitochondria, nuclear factor erythroid 2-related factor 2

## Abstract

Diabetic bladder dysfunction (DBD) afflicts nearly half of diabetic patients, but effective treatment is lacking. In this study, IR-61, a novel heptamethine cyanine dye with potential antioxidant effects, was investigated to determine whether it can alleviate DBD. Rats were intraperitoneally injected with IR-61 or vehicle after diabetes was induced with streptozotocin. Before evaluating the effects of IR-61 in improving DBD by filling cystometry, we detected its distribution in tissues and subcellular organelles by confocal fluorescence imaging. Near infrared (NIR) imaging showed that IR-61 could accumulate at high levels in the bladders of diabetic rats, and confocal images demonstrated that it was mainly taken up by bladder smooth muscle cells (BSMCs) and localized in mitochondria. Then, filling cystometry illustrated that IR-61 significantly improved the bladder function of diabetic rats. The histomorphometry results showed that IR-61 effectively mitigated the pathological changes in bladder smooth muscle (BSM) in diabetic rats. Furthermore, IR-61 remarkably reduced the number of apoptotic BSMCs and the unfavorable expression of proteins related to the mitochondrial apoptotic pathway (Bcl-2, BAX, Cytochrome C, and cleaved Caspase-9) in diabetic rats. Moreover, the frozen section staining and transmission electron microscopy results proved that IR-61 significantly reduced the reactive oxygen species (ROS) levels and prevented the mitochondrial mass and morphology damage in the BSM of diabetic rats. In addition, IR-61 upregulated the expression of nuclear factor erythroid 2-related factor 2 (Nrf2) and its associated antioxidant proteins in the BSM of diabetic rats. Together, these results indicate that IR-61 can improve the voiding function of rats with DBD by protecting the mitochondria of BSMCs from oxidative stress, which is possibly mediated through the activation of the Nrf2 pathway.

## Introduction

The high incidence of urological complications associated with diabetes mellitus (DM), especially diabetic bladder dysfunction (DBD), has increasingly become a global public health concern (Arrellano-Valdez et al., 2014; Wessells et al., 2018). The symptoms of DBD include early urine storage problems, such as overactive bladder (OAB) with urgency incontinence, as well as late emptying disorders such as impaired bladder emptying, urinary retention, and overflow incontinence (Fayyad et al., 2009; Liu and Daneshgari, 2014; Wittig et al., 2019). Therefore, such different manifestations as overactive bladder and detrusor weakness may occur at different stages of DBD progression (Brown et al., 2005). Correspondingly, the pathogenesis of DBD mainly involves multifactorial and time-dependent impairment of the detrusor or dominated nerves, urothelium, or their combination (Daneshgari et al., 2009; Yang et al., 2019). In the late stage of DBD, bladder smooth muscle is a main site of pathological changes leading to impaired bladder function (Wang et al., 2017). The current treatments for DBD vary with the progression of the disease. The first consideration in managing DBD should be glycemic control. Anticholinergic drugs and beta3-agonists are the first choice for patients presenting with early signs of DBD and overactive bladder symptoms. Unfortunately, conservative treatment of patients with low bladder detrusor activity is difficult, such as parasympathomimetics, which not only have little benefit, but also have side effects that may outweigh any benefit, so these drugs are rarely used. Surgical procedures are the last resort to eliminate the symptoms of late DBD, such as sacral neuromodulation, but its effect may still be unsatisfactory (Wittig et al., 2019). Therefore, it is necessary to develop novel and effective therapies.

Oxidative stress caused by hyperglycemia is strongly related to such diabetic complications as DBD, and diabetic patients exhibit increased cellular levels of reactive oxygen species (ROS) and DNA damage (Dandona et al., 1996; Brownlee, 2001; Giacco and Brownlee, 2010). In addition, some studies have shown that the oxidative damage of BSMCs may be a contributing factor to DBD (Beshay and Carrier, 2004; Andersson, 2018). With increased ROS production, four pathways are activated, namely, polyol flux, intracellular advanced glycosylation end product (AGE) formation, protein kinase C activation, and hexosamine pathway flux (Giacco and Brownlee, 2010). The impact of mitochondrial function in this process cannot be ignored. Briefly, the intracellular free radicals induced by hyperglycemia disrupt the redox homeostasis maintained by mitochondria, and mitochondrial damage also produces a large amount of ROS, which exacerbate the pathological changes (Towler, 2013; Koliaki and Roden, 2016). Mitochondrial dysfunction also leads to a decrease in ATP production, alterations in cellular function and structure, and loss of organ function (Bhargava and Schnellmann, 2017; Bertero and Maack, 2018). Thus, the mitochondrial oxidative stress-targeted therapeutics might be beneficial for patients with DBD.

The balance of ROS levels is tightly controlled by regulation of the production and elimination of endogenous antioxidants that act as scavengers under physiological conditions. Nrf2, one of the most well-studied inducible transcription factors, regulates antioxidant response element-mediated expression of detoxifying and antioxidant enzymes, such as heme oxygenase-1 (HO-1), to protect against oxidative stress (Chu et al., 2017; Battio et al., 2018). Previous studies have indicated that activation of the Nrf2 pathway can effectively inhibit bladder tissue damage (Liu et al., 2016; Gu et al., 2018), including DBD (Lin et al., 2019). In a previous study, we synthesized IR-61, a small molecule near-infrared (NIR) fluorescent heptamethine cyanine dye that exhibits good biocompatibility and results in minimal cellular contamination to the microenvironment. IR-61 preferentially accumulates in mitochondria and alleviates the cell damage caused by oxidative stress (Wang et al., 2016). However, whether IR-61 can protect bladder function has not been determined. In the present study, we investigated the effects of IR-61 on bladder function and the pathological alterations of BSM in diabetic rats as well as the underlying mechanism.

## Materials and Methods

### Reagents and Materials

The synthesis of IR-61 followed a previously reported method (Wang et al., 2016). The synthesized IR-61 powder was dissolved in dimethyl sulfoxide (DMSO) to a concentration of 10 mmol in advance and then stored at −20°C for subsequent use. Before each administration, we diluted the solution by 40-fold with sterilized PBS solution and mixed it well. All the above operations were performed in the dark. Streptozotocin (STZ) was purchased from Sigma-Aldrich (United States).

### Animal Preparation

The experiments were conducted on female Sprague Dawley rats (weighing 180–220 g) that were purchased from the animal facility of the Central Animal House Services of the Army Medical University (AMU, Third Military Medical University), Chongqing, China. All protocols and animal research procedures were approved by the Ethics Committee and performed in accordance with the guideline of the Animal Care and Use Committee of the Third Military Medical University. The rats were housed at room temperature and exposed to a 12-h/12-h light–dark cycle, with free access to food and water. All the rats were randomly divided into two groups: CON (control rats, *n* = 15) and DM (diabetic rats, *n* = 30). After 12-h fasting, the rats in the DM group were intraperitoneally injected with 65 mg/kg STZ that was dissolved in citrate-sodium citrate buffer (0.05 M, pH 4.0–4.5). The rats in the Control group were treated with an equal volume of vehicle (0.05 M citric acid-sodium citrate buffer, pH 4.0–4.5). Blood glucose (GLU) was measured *via* a glucometer (Roche Diagnostics Corporation, Indianapolis, IN, United States) using blood sampled from the tail vein 3 d and 7 d after injection. The rats with GLU levels above 16.7 mmol/L on both days were considered diabetic and selected for the subsequent experiments. The rats in the DM group were randomly divided into two groups: rats with diabetic bladder dysfunction treated with vehicle (DBD; *n* = 15) and rats with diabetic bladder dysfunction treated with IR-61 (DBD + IR-61; *n* = 15). IR-61 (1.6 mg/kg) was injected intraperitoneally once a week, and the administration duration was 10 weeks. The dose of 1.6 mg/kg showed significant protective effects on rats with DBD, which has been demonstrated in our previous experiments. Blood glucose and body weight were also measured every week.

### *Ex Vivo* and *In Vitro* NIR Imaging of IR-61

The diabetic rats were intraperitoneally injected with IR-61 (1.6 mg/kg) and then sacrificed under anesthesia. Their hearts, livers, spleens, lungs, kidneys, and bladders were harvested for *ex vivo* imaging at 6 h, 1 days, 4 days, and 7 days after injection. At each time point, three diabetic rats were used for detection. Near-infrared fluorescence imaging was performed to detect the distribution of IR-61 in the organ using the Kodak FX Professional Imaging System (New Haven, CT). To detect the distribution of IR-61 in the bladder, the bladder tissues were frozen and cut into slices (8 μm). The fluorescence signals of IR-61 were detected using Leica LAS AF Lite software (Leica, Weztlar, Germany).

To determine the subcellular location of IR-61 in BSMCs, primary BSMCs were isolated from the SD rats and cultured with an adherent cultivation approach, and immunofluorescence staining with Gt anti-α-SMA antibody (1:100, ab21027, Abcam) was performed to identify the cells as previously described (Gopinath et al., 2013). The cultured BSMCs were then seeded in a 35-mm petri dish and incubated with 10 mM IR-61 for 20 min and 100 nM Mito-Tracker Green (M7514, Invitrogen) for 30 min. The cell nuclei were stained with Hoechst 33342 (C1025, Beyotime, China) and photographed with a confocal microscope (Leica TCS SP5).

### Cystometry *In Vivo*

For *in vivo* repetitive cystometry analysis, the rats treated for 10 weeks were anesthetized (Brouwers et al., 2013; Funahashi et al., 2014; Magaldi et al., 2019), placed in a supine position on top of a thermal pad, and fixed on a small animal operating table with an elastic bandage. Then, the bladder was surgically exposed *via* a midline abdominal incision, and a PE-50 catheter was inserted through the apex of the bladder dome. The other end of the catheter was connected to a pressure transducer (Laborie Medical Technologies Inc., Beijing, China) *via* a three-way stopcock to record the intravesical pressure and a micro medicine infusion pump (Hangzhou Zeda Instruments Co., Ltd., Hangzhou, China) to infuse saline into the bladder. After the bladder was emptied, sterile room-temperature saline was infused through the micro medicine infusion pump at a rate of 0.3 ml per minute. Pumping was immediately stopped when urine was observed at the external urethra and started again when urination ended. The intravesical pressure curve was automatically recorded with a computer for 30 min. The *in vivo* cystometry test parameters included the urination frequency within 30 min, maximum bladder capacity (MBC, the volume of saline pumped before first urination), maximum voiding pressure (MVP, the maximum peak pressure during the actual voiding phase), residual volume (RV, manually drained and measured with a 2.5-ml syringe), voiding efficiency {VE, calculated as [(MBC-RV)/MBC] *100%}, and bladder compliance [BC, calculated as (MBC/TP-BP)]. Threshold pressure (TP) is defined as the intravesical pressure immediately before micturition. Basal pressure (BP) is defined as the minimum pressure between two micturition (Supplementary Figure S1). Five rats in each group were measured. The mean value of the three voiding cycles of each rat was used to eradicate any discrepancies. The procedure was performed three times for each rat (Wang et al., 2019).

### Histological Examinations

Rats in each group (*n* = 5) were euthanized after filling cystometry, and the bladders of the rats were isolated and fixed using 4% paraformaldehyde solution for 48 h at room temperature. After fixation, the tissues were dehydrated, embedded in paraffin, and sliced into 5-μm-thick sections for H&E and Masson’s trichrome staining. Sections were prepared for immunofluorescence staining; these sections were deparaffinized, rehydrated, and treated with 3% hydrogen peroxide for 10 min at room temperature to eliminate the endogenous peroxidase activity. Then, the sections were placed in 0.01 M citrate buffer solution (pH 0.6), boiled (95–100°C) for 15 min to retrieve the antigen, and subsequently blocked with 1% goat serum albumin for 30 min at room temperature. After incubation with primary antibodies against α-SMA (Abcam, 1:200) and Nrf2 (CST, 1:200) overnight at 4°C, the sections were incubated with the corresponding secondary antibodies for 1 h at 37°C. The stained bladder sections were examined under a light microscope (Olympus Inc., Tokyo, Japan), and images were captured with a digital camera mounted to the microscope. All the images were analyzed using ImageJ software.

### TUNEL Assay to Detect the Apoptosis of BSMCs

An *in situ* cell death detection kit, Fluorescein (11684817910, Roche Applied Science, Indianapolis, IN, United States), was used to detect the apoptosis of BSMCs according to the manufacturer’s instructions. Briefly, the bladder tissue sections in each group (*n* = 5) were hydrated and treated with a proteinase K working solution at 37°C for 15 min. Next, the sections were incubated with the TUNEL reaction mixture (the negative control group was only treated with 50 μl of fluorescein-labeled dUTP solution) in a dark, wet box at 37°C for 1 h. Finally, DAPI (Sigma-Aldrich) was used to stain the nuclei. The stained sections were photographed using a fluorescence microscope.

### *In Situ* Detection of Mitochondria and Superoxide Levels

Fresh bladder tissues in each group (*n* = 5) were prepared into frozen slices and incubated quickly with Mito-Tracker Red, 2′,7′-dichlorodihydrofluorescein diacetate (DHE, 10 μm, Beyotime) and MitoSOX™ Red (10 μm, Invitrogen) at 37°C in the dark for 30 min to measure the relative number of active mitochondria, the *in situ* level of intracellular ROS, and the level of mitochondrial superoxide in the BSMCs, respectively. Quantitative analysis of the fluorescence intensity was performed using ImageJ software.

### Transmission Electron Microscopy

Fresh BSM tissues in each group (*n* = 5) trimmed into 1*1*3 mm tissue blocks were fixed in 4% glutaraldehyde overnight, fixed with 1% osmium tetroxide, dehydrated in graded ethanol and infiltrated in fresh 100% resin. Ultrathin sections cut from the blocks were stained with 5% uranyl acetate and lead citrate and then viewed by transmission electron microscopy (JEM-1400PLUS, Japan) at 100 KV.

### Western Blotting Analysis

Bladder tissues in each group (*n* = 5) that were denuded of mucosal and adventitial layers were homogenized and lyzed on ice for 30 min with RIPA buffer (Beyotime Biotechnology, China) containing a cocktail of protease and phosphatase inhibitors (Thermo Scientific, IL, United States). After centrifugation (15,000 g at 4°C for 15 min), the supernatants were collected, and the protein concentration was measured using a BCA protein assay kit (Beyotime Biotechnology, China). Samples containing equivalent amounts of proteins (20 mg) were separated by electrophoresis on 10% SDS-polyacrylamide gels and transferred to polyvinylidene difluoride (PVDF) membranes (Millipore, United States)that were blocked with western blocking buffer (Beyotime Biotechnology, China). Then, the PVDF membranes were probed with primary antibodies at 4°C overnight, followed by incubation with horseradish peroxidase-conjugated secondary antibodies (CST, 1:1,000) for 1 h at room temperature. The band intensities were visualized and analyzed using an enhanced chemiluminescence detection system (Bio-Rad Laboratories) and ImageJ software (Bethesda, MD, United States). The primary antibodies used were ɑ-smooth muscle actin (α-SMA) (Abcam, 1:1,000), cleaved-Caspase 3 (Abcam, 1:1,000), Bax (1:1,000, Abcam), Bcl-2 (1:1,000, Abcam), cleaved-Caspase 9 (Abcam, 1:1,000), Cytochrome C (Abcam, 1:1,000), Nrf2 (CST, 1:1,000), Keap1 (CST, 1:1,000), SOD-1 (Abcam, 1:1,000), SOD-2 (Affinity, 1:1,000), HO-1 (CST, 1:1,000), GPX-1 (CST, 1:1,000), β-actin (1:1,000, Proteintech) and GAPDH (1:1,000, Beyotime Biotechnology, China).

### Statistical Analysis

All the data are presented as the mean ± SD. The statistical analyses were performed using one-way or two-way analysis of variance to determine statistical significance using SPSS 26.0 (SPSS Inc., Chicago, IL, United States). *p* < 0.05 was considered statistically significant.

## Results

### Biodistribution and Accumulation of IR-61

**FIGURE 1:**
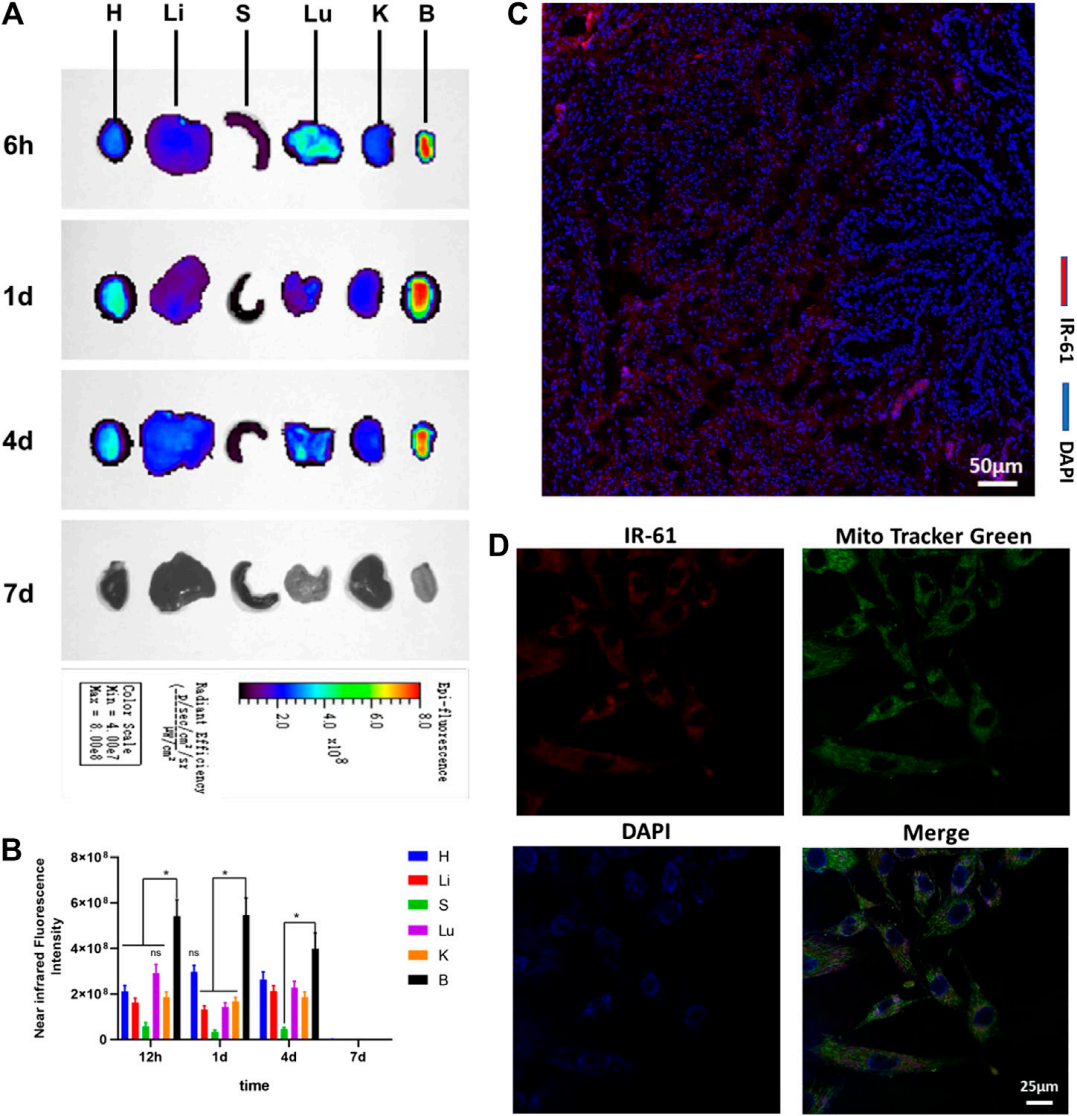
Biodistribution and subcellular localization of IR-61. **(A)** *Ex vivo* NIR imaging of vital rat organs 6 h, 1 days, 4 days and 7 days after intraperitoneal injection. H, heart; Li, liver; S, spleen; Lu, lung; K, kidney; B, bladder. **(B)** Quantitative analysis of NIR fluorescence intensities of vital organs at the indicated time points. **(C)** Fluorescence imaging of IR-61 (red) in bladder tissues, scale bar = 50 μm. **(D)** Determining the mitochondrial targeting of IR-61 in BSMCs by costaining with IR-61 and Mito-Tracker Green. Scale bar = 25 μm *p < 0.05 vs. NIR fluorescence intensities of the bladder.

Some vital organs were harvested from the diabetic rats for *ex vivo* imaging. We observed clearly stronger NIR fluorescence in the bladder than in other organs, such as the heart, liver, spleen, lung, and kidney. The results illustrated that IR-61 could highly accumulate in the bladder, as proven by the NIR fluorescence intensity of the bladder, which was significantly higher than that of other organs 6 h, 1 days, 4 days, and 7 days after intraperitoneal injection (Figure 1A). Imaging on 7 days after injection showed that there was no significant accumulation of IR-61 in the bladder, which is why we chose one injection per week as the frequency of administration.

To determine the tissue distribution of IR-61, fresh bladders were frozen and sectioned for histological analysis 1 day after IR-61 injection. Fluorescence microscopy showed that IR-61 was mostly localized in the smooth muscle layer of the bladder (Figure 1C and Supplementary Figure S2). Consequently, exploring the role of IR-61 in BSMCs was the focus of this study.

To define the subcellular location of IR-61, we isolated and cultured primary BSMCs from 8-week-old SD rats. The cells were identified by immunofluorescence staining of α-SMA, which is a specific marker of smooth muscle cells (SMCs) (Supplementary Figure S3). The targeted accumulation of IR-61 in the mitochondria of BSMCs was confirmed by its colocalization with MitoTracker Green (Figure 1D and Supplementary Figure S4). These results support the claim that IR-61 is a mitochondria-targeting NIR dye, which is consistent with the previous study.

### General Characteristics of the Rats

Supplementary Figures S5A,B describe the changes in the body weight and blood glucose levels of the rats in the three groups. Compared with the control group, the DBD group exhibited remarkably reduced body weight (*p* < 0.0001) and increased blood glucose levels (*p* < 0.0001) at week 10. No significant differences in these two parameters were observed between the DBD and DBD + IR-61 groups (Table 1). The results described above indicated that IR-61 did not change the blood glucose levels or body weights of the diabetic rats.

**TABLE 1:** Changes in body weight and blood glucose levels in each group.

	Pre-DBD induction	5 W	10 W
Body weight (g)			
Control (*n* = 10)	210.4 ± 5.420	293.0 ± 14.48	313.4 ± 8.080
DBD (*n* = 10)	213.5 ± 9.071	181.7 ± 29.56^****^	167.1 ± 27.77^****^
DBD + IR-61 (*n* = 10)	213.3 ± 9.499	201.9 ± 22.42^****^	201.3 ± 25.65^****^
Blood glucose (mg/dl)			
Control	6.386 ± 0.5014	6.600 ± 0.5447	6.157 ± 0.2225
DBD	6.329 ± 0.4348	33.086 ± 0.567^****^	33.229 ± 0.189^****^
DBD + IR-61	6.343 ± 0.4614	32.757 ± 0.872^****^	33.257 ± 0.113^****^

Data indicate the mean ± SD (****p < 0.0001 vs. control group).

The bladder weight was significantly increased in the DBD and DBD+IR-61 groups compared with the control group (Supplementary Figure S5C). However, the bladder weight was still significantly lower in the DBD+IR-61 group than in the DBD group. Since the development of the animals during the 10 weeks experiment differed, we further evaluated the ratio of the bladder weight to the body weight of the rats. Our results indicated that the ratio was significantly higher in the DBD and DBD + IR-61 groups than in the Control group (*p* < 0.0001, *p* < 0.001, respectively Supplementary Figure S5D). There was no difference between the DBD group and DBD + IR-61 group.

### IR-61 Protected the Bladder Function of the Diabetic Rats

**FIGURE 2:**
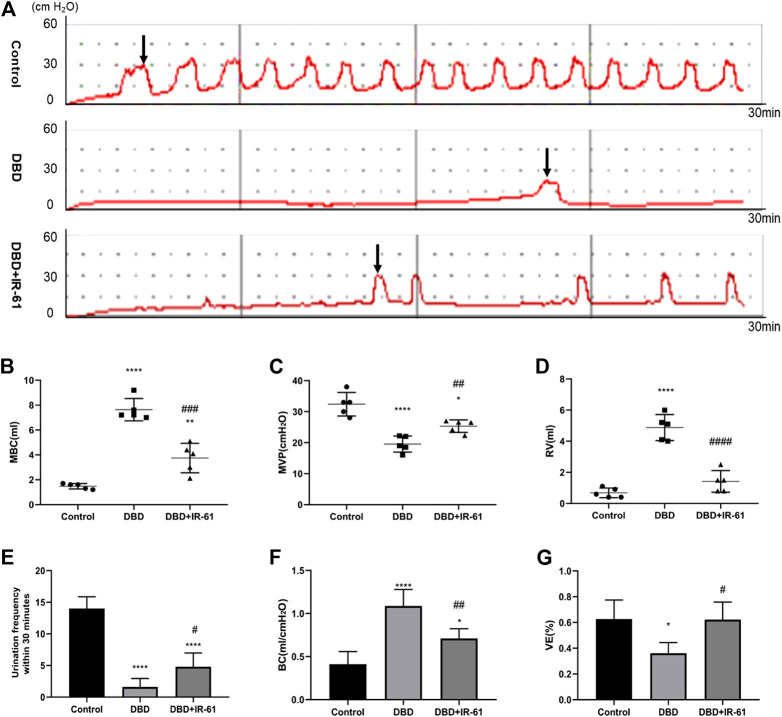
Cystometry results of all groups (n = 5). **(A)** Representative cystometry curves of rats in each group. **(B)** Maximum bladder capacity (MBC) **(C)** Maximum voiding pressure (MVP). **(D)** Residual volume (RV) **(E)** Frequency of urination in 30 min (UF30 min). **(F)** Bladder compliance [BC; calculated as MBC/(TP-BP)]. **(G)** Voiding efficiency {VE, calculated as [(MBC−RV)/MBC] × 100%}. Data indicate the mean ± SD (*p < 0.05, **p < 0.01, ***p < 0.001, ****p < 0.0001 vs. control group; #*p* < 0.05, ##*p* < 0.01, ###*p* < 0.001 vs. DBD group). Black arrows indicate the micturition peaks.

The representative cystometry curves of each group are presented in Figure 2A. The analysis results of the curves revealed that the rats in the DBD group had significantly decreased MVP, urination frequency, and VE (vs. Control *p* < 0.0001, *p* < 0.0001, *p* < 0.05, respectively) but increased MBC, RV, and BC (vs. Control *p* < 0.0001 for each), suggesting typical DBD in the decompensation phase (Figures 2B–G); however, IR-61 treatment remarkably increased the MVP, urination frequency, and VE (vs. DBD group *p* < 0.01, *p* < 0.05, *p* < 0.05, respectively) but significantly decreased the MBC, RV, and BC (vs. DBD group *p* < 0.001, *p* < 0.0001, *p* < 0.0001, respectively). These data confirmed that IR-61 protected the voiding function of the diabetic rats.

### IR-61 Prevented Bladder Histological Changes Caused by Diabetes

**FIGURE 3:**
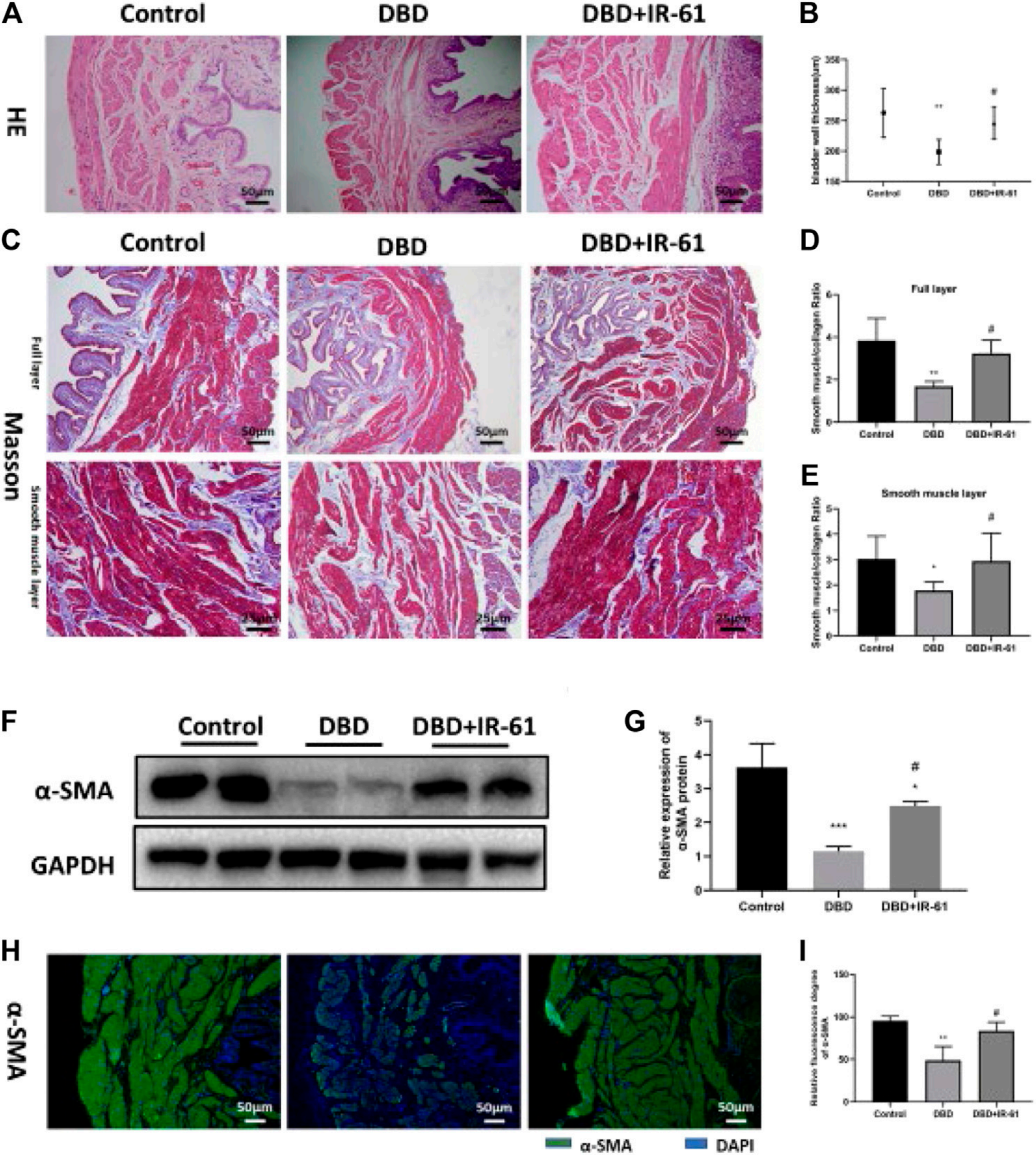
IR-61 prevented DM-induced histopathological changes. **(A)** Representative HE staining images of bladder tissues from each group. **(B)** Bladder wall thickness (BWT). Scale bar = 50 μm. BWT measured from HE images. **(C)** Representative Masson’s trichrome staining images of bladder tissues from each group. **(D,E)** Bladder smooth muscle to collagen ratio in the entire bladder wall and smooth muscle layer determined by Masson’s trichrome images. **(F**,**G)** Levels of α-SMA in bladder tissues detected by Western blot. **(H**,**I)** Levels of α-SMA in bladder tissues detected by immunofluorescence, scale bar = 50 μm. Data indicate the mean ± SD (*p < 0.05, ****p < 0.0001 vs. control group; #*p* < 0.05, ##*p* < 0.01 vs. DBD group).

The bladder wall thickness was measured using H&E images (Figure 3A), and the ratio of smooth muscle to collagen in the full layer and smooth muscle layer was determined by Masson’s trichrome staining (Figure 3C). Morphometric analysis of bladder tissues revealed that the bladder wall thickness and the smooth muscle/collagen ratio in the full layer and smooth muscle layer were significantly decreased (*p* < 0.01, *p* < 0.01, *p* < 0.05, respectively) in the rats in the DBD group, but IR-61 treatment prevented the decrease of bladder wall thickness and the smooth muscle to collagen ratio (*p* < 0.05 for each) (Figures 3B,D,E); these results indicated that IR-61 could effectively alleviate the bladder tissue damage in the diabetic rats.

In addition, we detected the expression of α-SMA in bladder tissue by WB and immunofluorescence staining and found that the expression of α-SMA was decreased in the DBD group (vs. Control *p* < 0.05) and increased in the DBD + IR-61 group (vs. DBD group *p* < 0.05), which also indicated that IR-61 could prevent the decreased smooth muscle content caused by DM.

### Ir-61 Reduced the Apoptosis of Bladder Smooth Muscle Cells

**FIGURE 4:**
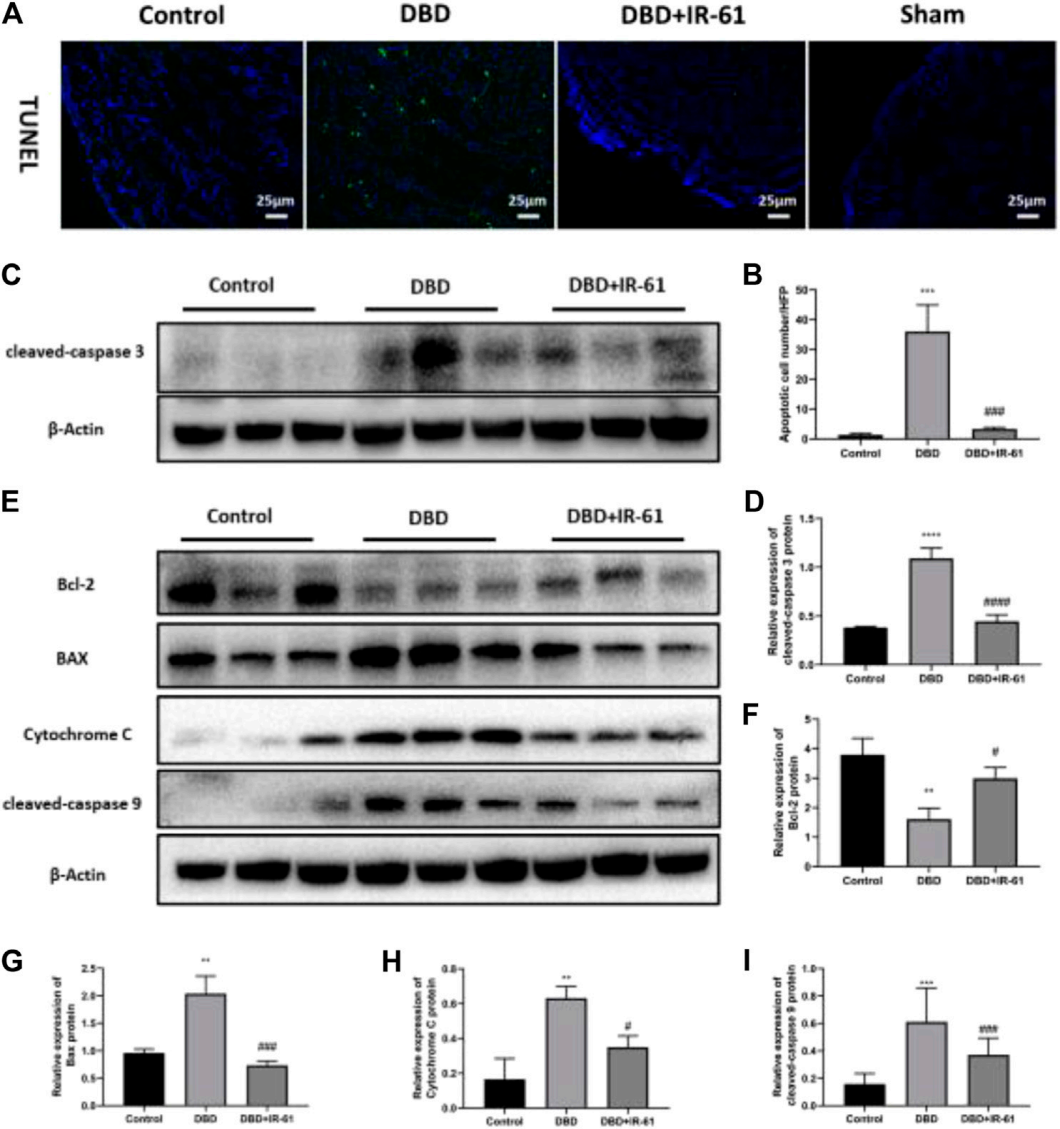
IR-61 reduced number of apoptotic BSMCs in DM and affected mitochondrial apoptosis-related proteins. **(A)** Representative images of TUNEL staining (green) in bladder. **(B)** Number of apoptotic BSMCs per high-power field. **(C,D)** Levels of cleaved-Caspase three in bladders detected by Western blot. **(E–I)** Bcl-2, BAX, Cytochrome C, and cleaved-caspase nine expression levels in each group and quantitative analysis. Data indicate the mean ± SD (*p < 0.05, **p < 0.01, ***p < 0.001 vs. control group; #*p* < 0.05, ###*p* < 0.001 vs. DBD group).

We examined whether IR-61 had any protective effects on the number of apoptotic BSMCs. TUNEL staining demonstrated that the number of apoptotic cells in the bladder was markedly increased in the DBD group compared with the Control and DBD + IR-61 groups (*p* < 0.001 for each, Figures 4A,B). Moreover, Western blotting showed that the protein level of cleaved Caspase-3 was clearly increased in the DBD group and markedly decreased in the DBD + IR-61 group compared with the control group (*p* < 0.001 for each, Figures 4C,D). These data illustrated that IR-61 ameliorated the apoptosis of BSMCs.

To determine whether the apoptosis of BMSCs and the anti-apoptotic effect of IR-61 in diabetic rats are related to mitochondria, we conducted Western blot assays on proteins associated with mitochondrial apoptosis pathways, such as Bcl-2, BAX, Cytochrome C, and cleaved Caspase-9 (Figures 4E–I). The results indicated that the protein levels of BAX, Cytochrome C, and cleaved Caspase-9 were significantly increased (*p* < 0.01 for each) and the protein levels of Bcl-2 were decreased (*p* < 0.05, *p* < 0.001, *p* < 0.05, *p* < 0.05, respectively) in the DBD group compared with the Control group, but IR-61 treatment prevented these changes. All these data demonstrated that IR-61 inhibited mitochondrial-related cellular apoptosis in the bladder tissue of the diabetic rats.

### IR-61 Mitigated the Mitochondrial Damage and ROS Production in the Diabetic Bladders

**FIGURE 5:**
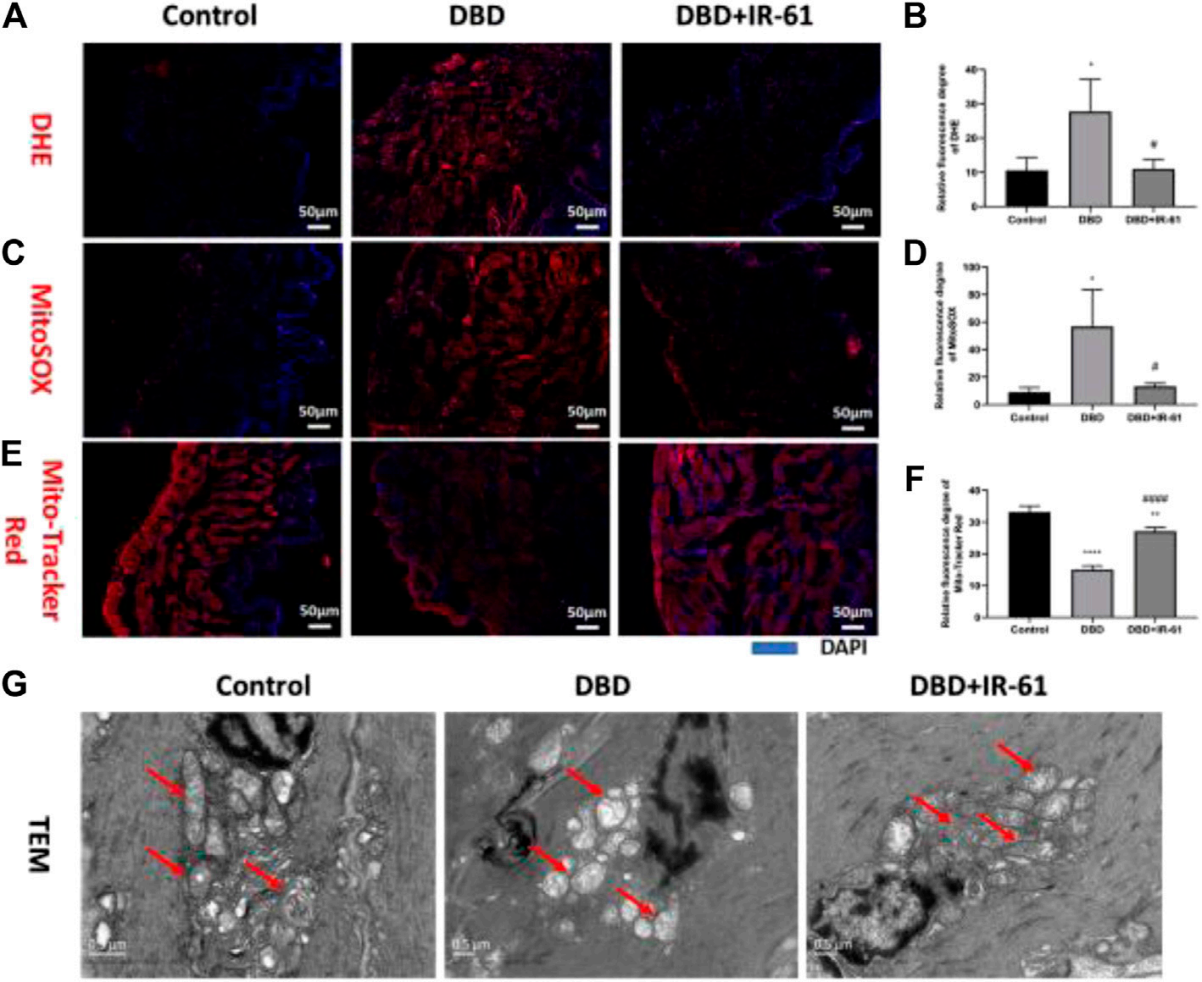
IR-61 alleviated diabetes-induced oxidative stress in bladder tissues and damage to mitochondria in BSMCs. **(A,B)** Intracellular ROS in bladders detected by DHE staining of frozen bladder sections, scale bar = 50 μm. **(C,D)** Mitochondrial ROS in bladders detected by MitoSOX staining of frozen bladder sections, scale bar = 50 μm. **(E,F)** Mitochondrial mass of BSMCs measured by Mito-Tracker Red staining of frozen bladder tissue sections, scale bar = 20 μm. **(G)** Representative transmission electron micrographs of mitochondria morphology and structure in BSMCs. Red arrows indicate mitochondria, scale bar = 0.5 μm. Data indicate the mean ± SD (*p < 0.05, **p < 0.01, ***p < 0.001 vs. control group, #*p* < 0.05, ###*p* < 0.001 vs. DBD group).

Frozen bladder tissue sections were stained with dihydroethidium (DHE) (Figure 5A) and MitoSOX (Figure 5C) and used to measure the cellular and mitochondrial superoxide levels. The results of analyzing the DHE and MitoSOX images showed that both the cellular and mitochondrial superoxide levels were clearly increased by DBD (*p* < 0.05 for each) and suppressed by IR-61 treatment (*p* < 0.05 for each); these results revealed the antioxidative effect of IR-61 (Figures 5B,D). Moreover, the fluorescence staining of MitoTracker Red (Figure 5E) proved that IR-61 preserved mitochondrial function, which was significantly reduced in bladder tissues of the DBD group (*p* < 0.0001, Figure 5F). Transmission electron microscopy (TEM) images of BSM showed that the BSMCs in the rats in the DBD group exhibited increased mitochondrial calcification and degeneration, whereas IR-61 attenuated the damage to BSMC mitochondria observed in the diabetic rats (Figure 5G); these results showed that IR-61 had a protective effect on the mitochondria of BSMCs.

### IR-61 was Involved in the Activation of the Nrf2 Pathway

**FIGURE 6:**
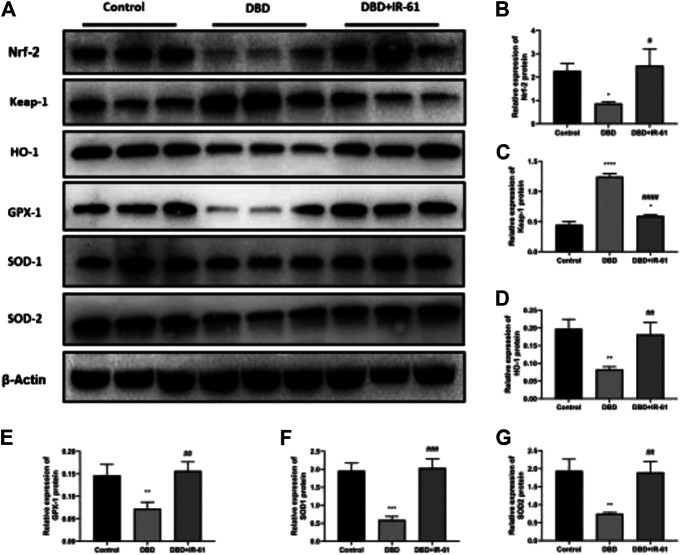
IR-61 activated Nrf2 and increased antioxidant-related protein levels. **(A–G)** Western blot analysis of Nrf2, Keap1, HO-1, GPX-1, SOD-1, and SOD-2 in bladder tissue and quantitative analysis. Data indicate the mean ± SD (*p < 0.05, **p < 0.01, ***p < 0.001, ****p < 0.0001 vs. control group, #*p* < 0.05, *p* < 0.01, ###*p* < 0.001, ####*p* < 0.0001 vs. DBD group).

As shown in Figure 6, we explored whether the antioxidative stress effect of IR-61 is due to the activation of Nrf2. Representative original blots of the protein levels in the bladders of the three groups were examined (Figure 6A). Compared with those in the control group, the protein levels of Nrf2, HO-1, GPX-1, SOD-1, and SOD-2 were decreased in the DBD group (*p* < 0.05, *p* < 0.05, *p* < 0.01, *p* < 0.001, *p* < 0.01, respectively, Figures 6B,D–G), while the protein levels of Keap1 were increased (*p* < 0.0001, Figure 6C). However, IR-61 treatment significantly prevented the DM-induced reduction in Nrf2, HO-1, SOD-1, SOD-2 and suppressed the increase of Keap-1 observed in the bladders in the DBD + IR-61 group, which indicated that the protective effect of IR-61 may be related to the activation of Nrf2.

## Discussion

Diabetic bladder dysfunction has increasingly become a concern for those affected by DM (both type I and II). The clinical manifestations of DBD can vary and are extremely burdensome to patients, affecting their quality of life (Yuan et al., 2015; Wittig et al., 2019). However, there is still no sufficiently effective clinical treatment for DBD, and most of the current treatments only aim to relieve the symptoms of patients (Gomez et al., 2011). Hence, there remains an urgent need to develop effective medicines to preserve normal bladder function and to prevent or slow the progression of DBD. We have previously reported that IR-61, a heptamethine cyanine dye, significantly alleviated the cell damage caused by acute oxidative stress in cell therapy (Wang et al., 2016). In the present study, we found that IR-61 was able to accumulate in diabetic bladder tissue and prevented bladder dysfunction (Figure 2) through its antioxidative (Figures 5, 6) and antiapoptotic effects (Figure 4); these findings suggested that IR-61 may be a potential candidate for the treatment of DBD.

Before evaluating the effects of IR-61 in improving DBD, we found that IR-61 can accumulate in the bladder of diabetic rats and confirmed that it was localized in BSM and in the mitochondria of BSMCs. At the same time, we observed that IR-61 did not significantly improve the damage of diabetic bladder epithelial tissue in bladder histomorphological experiments (Supplementary Figure S6). Based on that, we decided to investigate the effects of IR-61 on BSM. BSM provides structural support for bladder contraction and relaxation, playing an essential role in maintaining normal bladder function. In the late stage of DBD, disturbance of the bladder detrusor, one of the main pathological changes, is attributed to different mechanisms, such as intercellular connection changes and excitability, muscarinic receptor density and distribution, intracellular signaling changes, and genetic changes (Wang et al., 2009; Masuda et al., 2020). Previous studies have shown that the activities of ion pumps, which are important modulators of bladder smooth muscle tone, were impaired in rats with STZ-induced diabetes (Mustafa, 2013), and BSM contractility was decreased in response to stimulation by potassium chloride and carbachol, which was associated with long-lasting hyperglycemia (Daneshgari et al., 2009). Another study found that hyperglycemia-induced oxidative stress activated pro-apoptotic pathways in BSMCs, eventually leading to bladder atony (Li and Oh, 2010). These findings prompted us to further investigate whether IR-61 can improve DBD by influencing BSM.

In our study, the urodynamic changes in the rats in the DBD group, including decreased VE and increased BC and RV, indicated that the diabetic rats had progressed to the stage of late decompensation, which was in accordance with the “temporal theory” of DBD (Klee et al., 2019; Majima et al., 2019; Yang et al., 2019). A decrease in the MVP values represents a decrease in bladder contractility, which indirectly demonstrates functional impairment of BSM. In addition, our histological examination verified the impairment of BSM in the rats with advanced DBD, including detrusor thinning and decreased smooth muscle to collagen ratio. We assumed that the histological changes described above may be due to the severe impairment of bladder voiding function in the decompensated stage such that the bladder was in a state of long-term urinary retention, resulting in continuous stress relaxation of BSM, followed by detrusor layer thinning, decreased smooth muscle/collagen and a series of changes in urination function. Furthermore, we found that there was a significant amount of apoptosis in the DBD group, which explained the reduced smooth muscle content in the bladder wall and decompensation in the context of hyperglycemia. It should be emphasized that the aforementioned apoptosis was related to the mitochondrial pathway, which was supported by our data (Figure 4). Interestingly, IR-61 treatment significantly prevented all the functional impairment and pathological changes mentioned above and downregulated the level of mitochondria-associated apoptosis in BSMCs.

In the late phase of DBD, chronic hyperglycemia is a more important cause of detrusor myogenic changes than polyuria (Xiao et al., 2013). Studies have verified that the cellular oxidative stress induced by hyperglycemia could cause alterations in lipids, proteins, and DNA, which ultimately may lead to organ dysfunction, such as DBD (Xiao et al., 2013; Inouye et al., 2018). As the major generator of ROS, mitochondria often become the target of elevated ROS levels, which leads to deadly consequences, such as oxidative damage to mitochondrial DNA (mtDNA) (Green et al., 2004; Sinha et al., 2013). In this experiment, we found that the total intracellular ROS and mitochondria-derived ROS levels in the bladder tissues of the diabetic rats were significantly increased compared with those in the control rats, but these levels were maintained by IR-61 treatment. Moreover, we observed significant mitochondrial destruction in the BSMCs of diabetic rats by transmission electron microscopy, but this impairment was prevented by IR-61 treatment. These data suggested that the damage to the bladder tissue in the diabetic rats may be due to sustained oxidative stress, which is consistent with previous studies. However, IR-61 reduced the level of oxidative stress in bladder tissues induced by a high-glucose environment and protected mitochondria from damage to maintain their normal function.

The Nrf2/Keap1 pathway has emerged as the most important transcription mechanism involved in regulating antioxidant genes and maintaining cellular redox homeostasis (Motohashi and Yamamoto, 2004; Cheng et al., 2011). Nrf2 is also involved in the regulation of mitochondrial homeostasis in diabetes by modulating mitochondrial biogenesis and membrane potential and could improve DBD in diabetic rats (Tomechko et al., 2015; Lin et al., 2019). Our data showed that the expression level of Nrf2 was significantly decreased in the diabetic rats, while IR-61 reactivated Nrf2 by downregulating Keap1. Subsequently, the expression of HO-1, GPX-1, SOD-1, SOD-2, and other relevant antioxidant proteins was increased, and this increased antioxidant protein expression could protect mitochondria and BSMCs from oxidative stress-mediated damage. In conclusion, IR-61 enhances cellular antioxidant and anti-apoptotic abilities by targeting mitochondria and regulating mitochondrial function, activating intracellular Nrf2/Keap-1 signaling pathways, followed by activating cellular endogenous antioxidant defense mechanisms and promoting the expression of a series of endogenous antioxidant proteins (such as GPX-1, SOD, etc.) (Wang et al., 2016).

In this study, we chose a STZ-induced type 1 diabetic rat model due to the higher incidence of DBD in patients with type 1 diabetes and the lower mean age of onset, which results in a more serious burden and greater suffering (Deli et al., 2013). In addition, there is no standard definition for the time point at which DBD progresses from the compensated stage to the decompensated stage. In a previous preliminary experiment, we found that rats STZ-induced diabetes already exhibited the urodynamic characteristics of decompensated DBD in the 10th week, which was consistent with other reports (Daneshgari et al., 2006; Klee et al., 2019; Yang et al., 2019). The limitation is that we did not verify the possible molecular mechanisms and conduct more in-depth studies.

**FIGURE 7:**
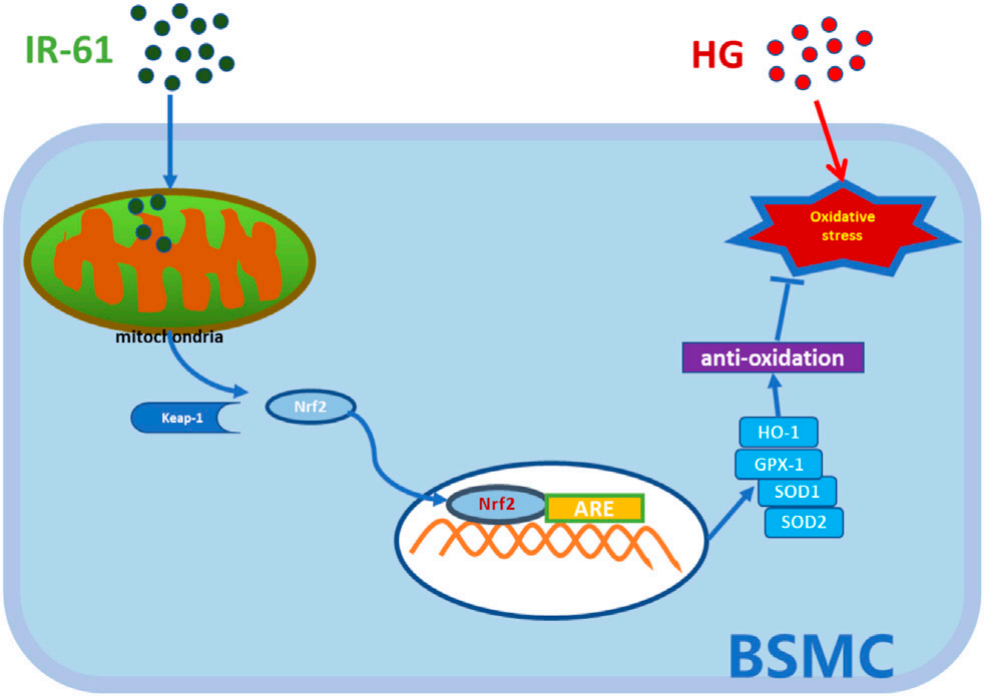
Illustration of the working hypothesis of the potential mechanism. The pre-administered IR-61 slightly elevates intracellular ROS level, which accelerates the dissociation of Nrf2 from Kelch-like ECH-associated protein-1 (Keap-1) into the nucleus *via* activating Nrf2 pathways. Increased Nrf2 nucleolus translocation elicits robust antioxidants, such as HO-1, SOD, and GPX-1, and then protects bladder smooth muscle cells (BSMCs) from oxidative stress damage induced by hyperglycemia, thereby preventing or slowing down the occurrence of bladder smooth muscle cells death.

In conclusion, our study showed that IR-61 protected the voiding function and prevented histological changes observed in diabetic rats and suppressed the apoptosis of BSMCs in the STZ-induced diabetic rat model. These effects might be related to mitochondrial protection via the Nrf2/Keap1 pathway rather than improved blood glucose levels (Figure 7). In summary, all these findings highlight the potential of IR-61 as a novel candidate preventive agent for DBD.

## Data Availability

The original contributions presented in the study are included in the article/Supplementary Material, further inquiries can be directed to the corresponding authors.
