# Supplementary material for: IR-61 Improves Voiding Function via Mitochondrial Protection in Diabetic Rats
Source: Front Pharmacol. 2021 Apr 14;12:608637. doi: 10.3389/fphar.2021.608637 (PMC8080033; doi:10.3389/fphar.2021.608637)
Supplement: Supplementary file 1 [file datasheet1.docx]

Supplementary Material

**Figure S1.** Bladder compliance (BC) = maximum bladder capacity/ (threshold pressure – basal pressure). Maximum bladder capacity (MBC) is defined as the volume of saline pumped before first urination. Threshold pressure (TP) is defined as the intravesical pressure immediately before micturition. Basal pressure (BP) is defined as the minimum pressure between two micturition.

**Figure S2.** The distribution of IR-61 in the bladder epithelium is few or even absent, and it is mainly distributed in the bladder smooth muscle layer. Scale bar=50 μm.

**Figure S3.** Identification of cultured BSMCs. Cultured BSMCs were identified by positive immunofluorescence staining with an anti-α-SMA antibody.

**Figure S4**. Determining the mitochondrial targeting of IR-61 in BSMCs by costaining with IR-61 and Mito-Tracker Green. Scale bar=10 μm.

**Figure S5.** The changes in body weight and blood glucose levels in rats in three groups. (A, B) Body weight and blood glucose levels of each group after STZ injection. (C, D) Bladder weight and bladder weight to body weight ratio of each group at 10 weeks. Data indicate the mean ± SD (**P < 0.01, ***P < 0.001, ****P < 0.0001 vs. control group, #P<0.05 vs. DBD group).

**Figure S6.** IR-61 has no obvious benefit on the bladder epithelial tissue of diabetic rats. Scale bar = 25μm.

**Figure S1**


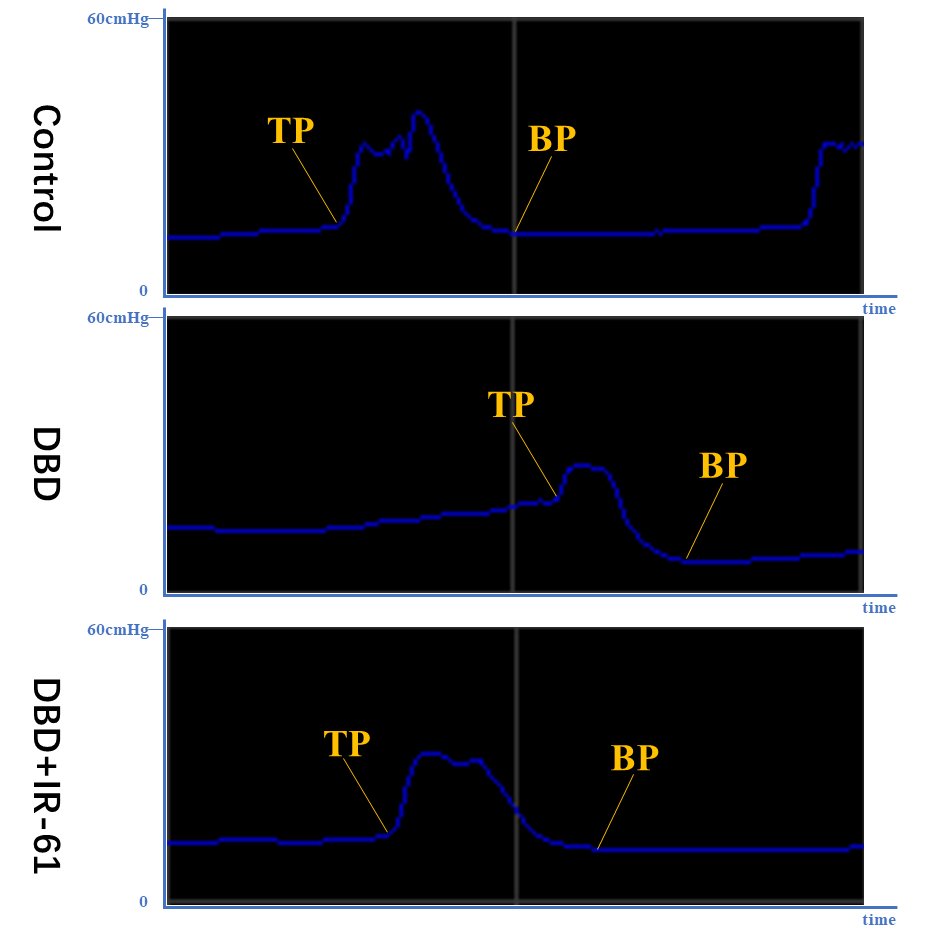


Figure S1. Bladder compliance (BC) = maximum bladder capacity/ (threshold pressure – basal pressure). Maximum bladder capacity (MBC) is defined as the volume of saline pumped before first urination. Threshold pressure (TP) is defined as the intravesical pressure immediately before micturition. Basal pressure (BP) is defined as the minimum pressure between two micturition.

**Figure S2**


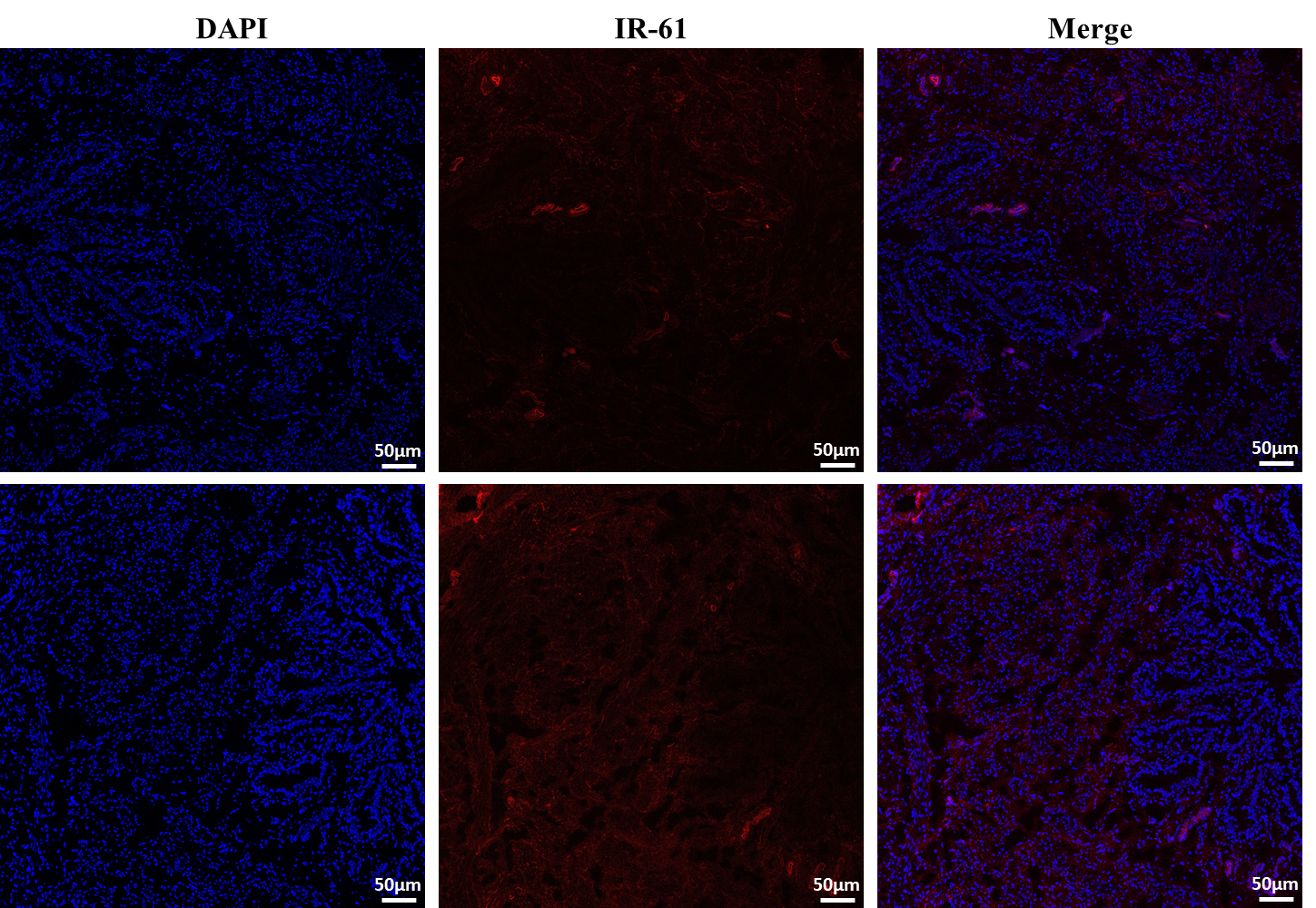


Figure S2. The distribution of IR-61 in the bladder epithelium is few or even absent, and it is mainly distributed in the bladder smooth muscle layer. Scale bar=50μm.

**Figure S3**


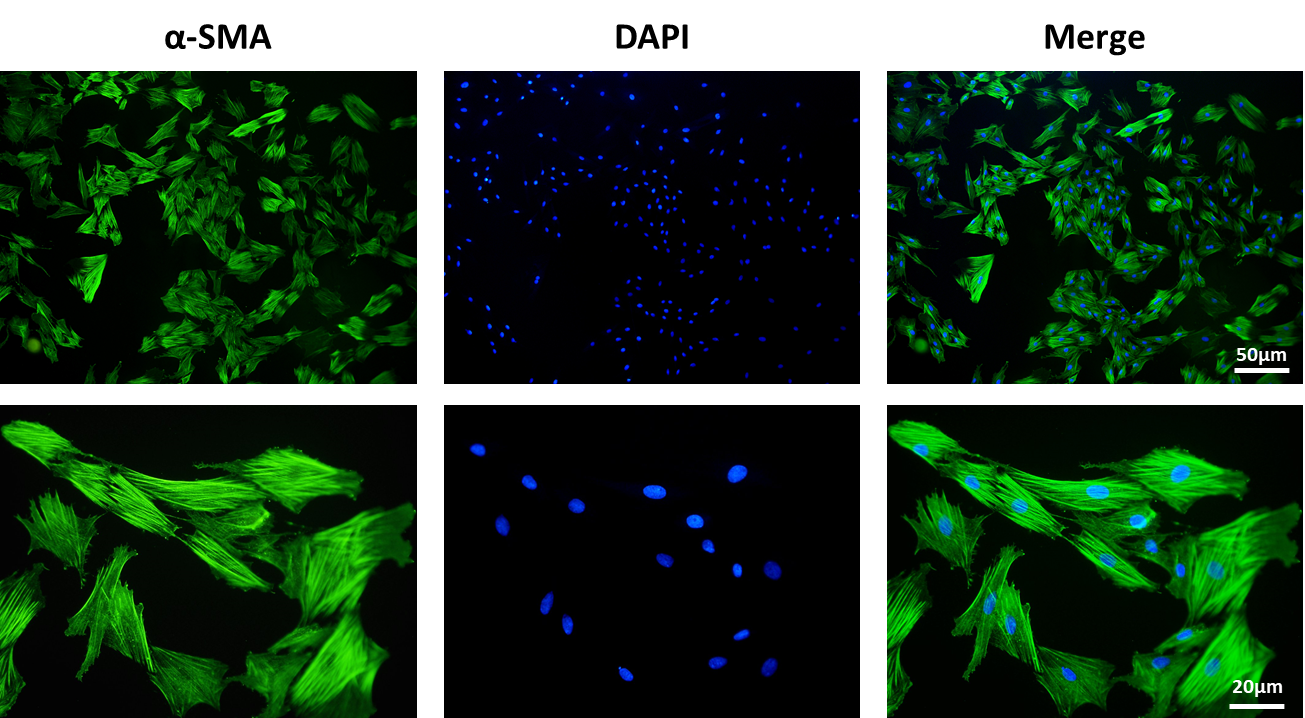


Figure S3. Identification of cultured BSMCs. Cultured BSMCs were identified by positive immunofluorescence staining with an anti-α-SMA antibody.

**Figure S4**

**
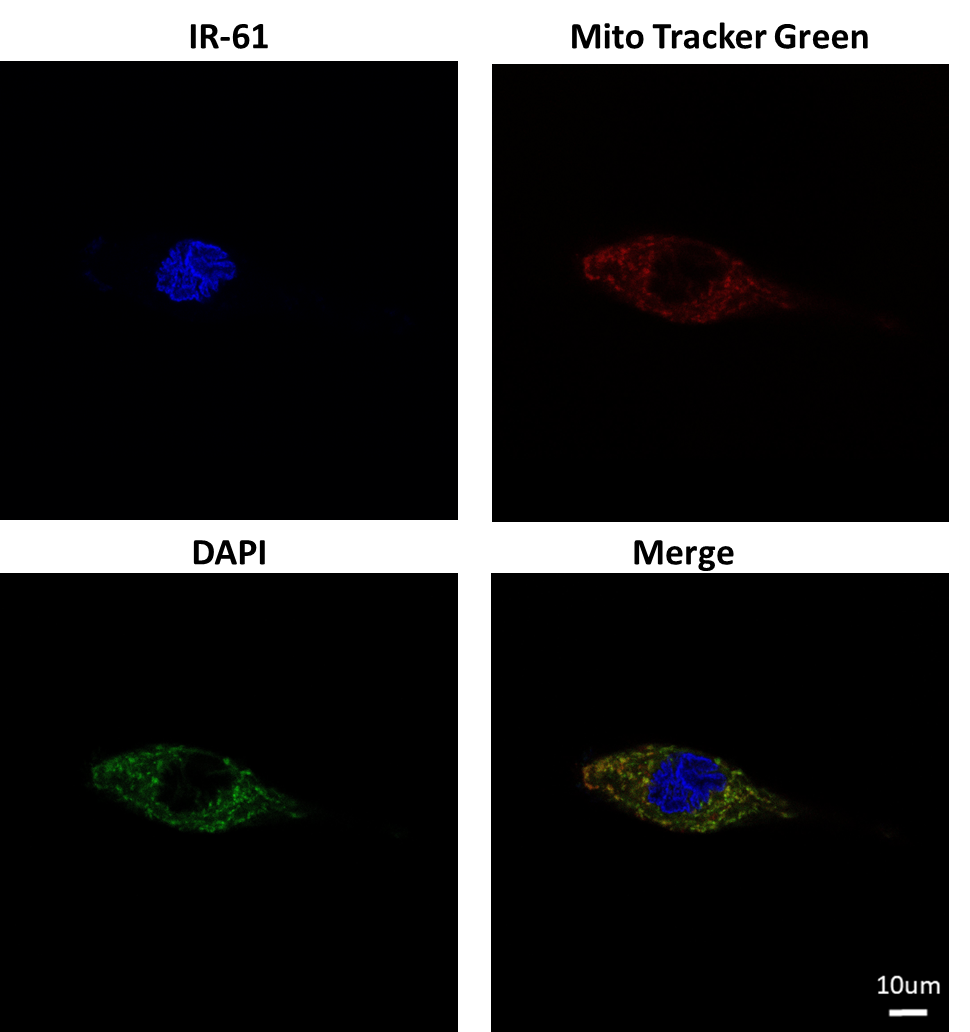
**

Figure S4. Determining the mitochondrial targeting of IR-61 in BSMCs by costaining with IR-61 and Mito-Tracker Green. Scale bar=10 μm.

**Figure S5**


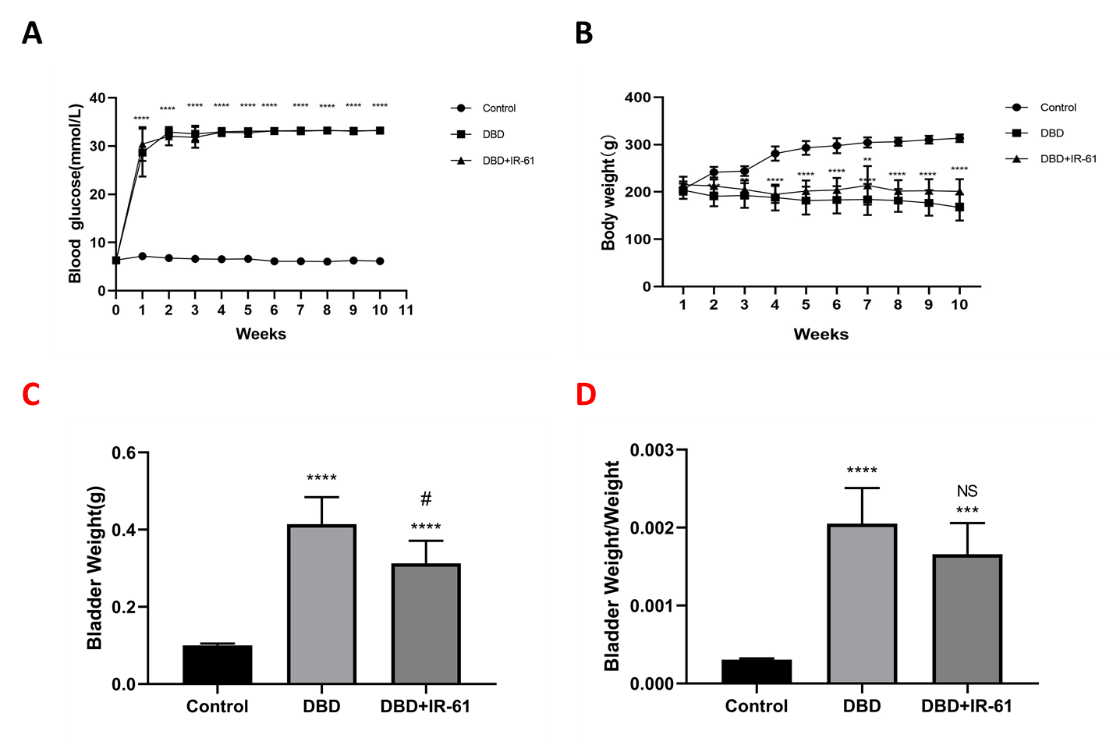


Figure S5. The changes in body weight and blood glucose levels in rats in three groups. (A, B) Body weight and blood glucose levels of each group after STZ injection. (C, D) Bladder weight and bladder weight to body weight ratio of each group at 10 weeks. Data indicate the mean ± SD (**P < 0.01, ***P < 0.001, ****P < 0.0001 vs. control group, #P<0.05 vs. DBD group).

**Figure S6**


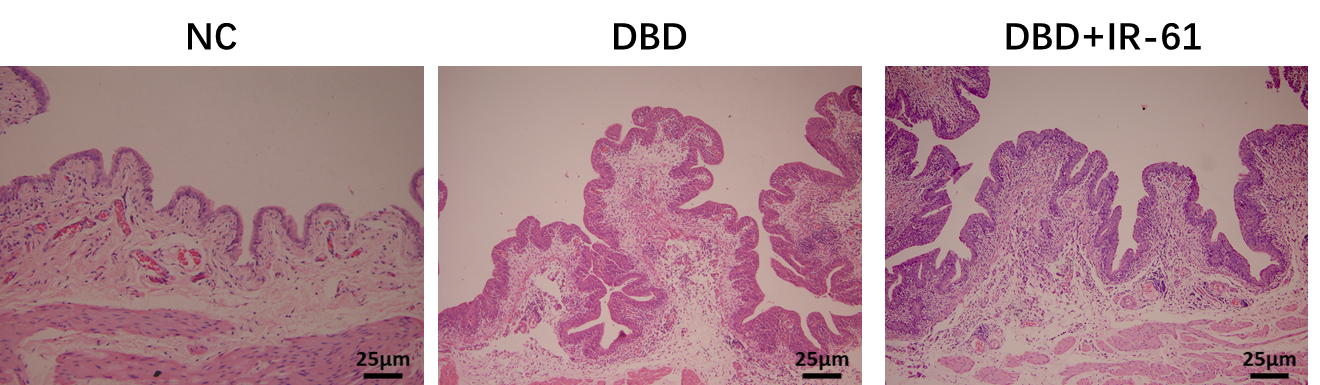


Figure S6. IR-61 has no obvious benefit on the bladder epithelial tissue of diabetic rats. Scale bar = 25μm.

**Figure S6**


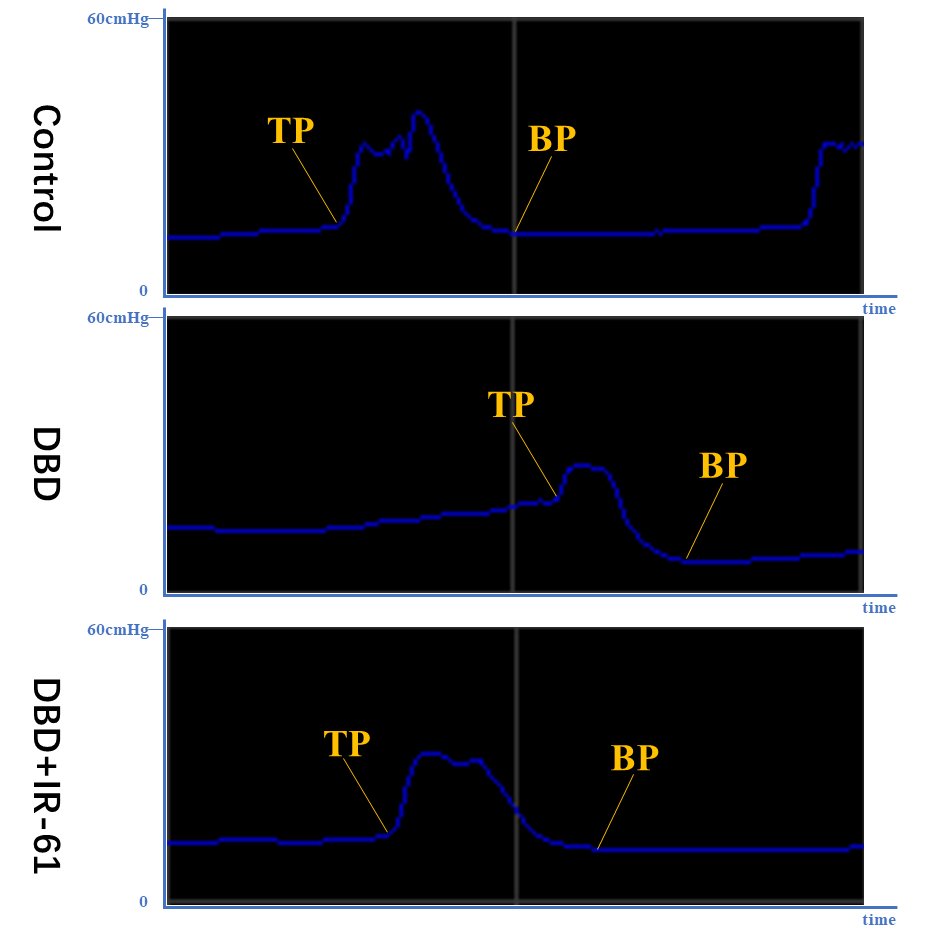


Figure S6. Bladder compliance (BC) = maximum bladder capacity/ (threshold pressure – basal pressure). Maximum bladder capacity (MBC) is defined as the volume of saline pumped before first urination. Threshold pressure (TP) is defined as the intravesical pressure immediately before micturition. Basal pressure (BP) is defined as the minimum pressure between two micturition.
